# A Case of Septicemia Due to Infection by Bacillus Dysenteriae Shiga

**Published:** 1918-05

**Authors:** 


***A Case of Septicemia Due to Infection by Bacillus Dysenteriae Shiga.*** By C. Muriel Astley Maer., att. R. A. M. C. *Brit. Med. Journ.*, Jan. 19, 1918.

The author reports a case of dysentery due to the Shiga bacillus, in which the organism was isolated from the blood and from the lesions of the large intestine at autopsy. The striking features of the case were the rapid progress of the disease, producing a profound toxemia caused by the septicemia; the marked cerebral symptoms, as compared with the clear mental condition exhibited in ordinary acute dysentery cases; the absence of any abdominal pain, tenderness, straining, or tenesmus. The author calls attention to the rarity of instances in which the Bacillus dysenteriae Shiga has been isolated from the blood.

